# Analysis of long-term results of pathogenetic treatment of Helicobacter pylori-associated gastroduodenopathies induced by nonsteroidal anti-inflammatory drugs in patients with osteoarthritis

**DOI:** 10.25122/jml-2020-0176

**Published:** 2021

**Authors:** Ludmila Mykhailivna Honcharuk, Olexander Ivanovicha Fediv, Svitlana Oleksandrivna Hresko, Antonina Anatoliivna Piddubna, Ludmila Viktorivna Mikulets, Ilona Tarasivna Rusnak, Dmytro Olexandrovich Hontsariuk, Yuliia Valeriievna Kokhaniuk

**Affiliations:** 1. Department of Internal Medicine and Infectious Disease, Bukovinian State Medical University, Chernivtsi, Ukraine; 2. Department of Gastroenterology, Municipal institution Regional Clinical Hospital, Chernivtsi, Ukraine; 3. Department of Clinical Immunology, Allergology and Endocrinology, Bukovinian State Medical University, Chernivtsi, Ukraine; 4. Department of Propaedeutics of Internal Medicine, Bukovinian State Medical University, Chernivtsi, Ukraine; 5. Department of Internal Medicine, Physical Rehabilitation and Sports Medicine, Bukovynian State Medical University, Chernivtsi, Ukraine

**Keywords:** osteoarthritis, nonsteroidal anti-inflammatory drugs, gastroduodenopathies, Helicobacter pylori, epidermal growth factor, tumor necrosis factor alpha, apoptosis, AHT – antihelicobacter therapy, EGF – epidermal growth factor, GDP – gastroduodenopathies, GIT – gastrointestinal tract, Hp – Helicobacter pylori, NSAIDs – nonsteroidal anti-inflammatory drugs, OA – osteoarthritis, PHP – practically healthy persons, TNF-α – tumor necrosis factor alpha

## Abstract

The study of the pathogenetic treatment and prevention of Helicobacter pylori (Hp)-associated gastroduodenopathies (GDP) induced by nonsteroidal anti-inflammatory drugs (NSAIDs) in patients with osteoarthritis (OA) is one of the most serious problems in modern clinical medicine. Sixty patients with OA and concomitant Hp-associated GDP induced by NSAIDs were examined. The levels of epidermal growth factor (EDF), sAPO-1/Fas and tumor necrosis factor-α (TNF-α) were determined. Group I included 30 patients who received triple anti-Helicobacter (AHT) therapy, and group II included 30 patients who received rebamipide. Long-term effects were assessed 6 months and 1 year after treatment. All subjects showed a significant increase in TNF-α (4.7 times), EDF (2.2 times) and a decrease in sAPO-1/Fas (3.6 times) levels compared to healthy individuals. After 1 month of treatment, a significantly more significant decrease in TNF-α and an increase in sAPO-1/Fas and EDF was found in group II. In the long-term treatment, a further decrease in TNF-α and an increase in the content of sAPO-1/Fas levels were observed in all groups. However, these changes were significantly more significant in group I compared to group I. The long-term follow-up showed a declining trend of EDF in all groups. The data obtained indicate the effectiveness of rebamipide in the complex pathogenetic treatment and prevention of Hp-associated GDP induced by NSAIDs in patients with OA.

## Introduction

Osteoarthritis (OA) is the most common disease of the musculoskeletal system, which contributes to the development of disability and a significant reduction in quality of life in people over 60 years [1]. Nonsteroidal anti-inflammatory drugs (NSAIDs) are essential in OA treatment due to their effective anti-inflammatory and analgesic effects. Every year, there are more than 500 million NSAIDs prescriptions in the world, 45 million being selective NSAIDs [2, 3]. However, the use of NSAIDs can lead to several injuries in the gastrointestinal tract (GIT), from the esophagus to the rectum. However, the lesion frequency is six times higher in the upper gastrointestinal tract compared to the lower [4, 5]. The risk of developing gastroduodenopathies (GDP) induced by NSAIDs does not depend on the duration of NSAID use. However, there is evidence of a slightly higher risk of enteropathy with long-term use of NSAIDs. In contrast, short-term use of these drugs increases the frequency of erosive-ulcerative lesions of the stomach.

Nonselective and selective NSAIDs cause intestinal damage with equal frequency. Literature data show that the incidence of severe gastrointestinal complications when taking selective NSAIDs remains 2.6 times higher than in patients not taking NSAIDs [6, 7].

Helicobacter pylori (Hp), along with NSAIDs, is a major etiological factor in the development of erosions and ulcers of the stomach and duodenum. According to a meta-analysis, Hp and NSAIDs are not independent risk factors but lead synergistically to gastrointestinal damage and bleeding [6].

The aim of the study was to establish the consequences of remote pathogenetic treatment based on the established patterns of Hp-associated GDP induced by NSAIDs in patients with OA.

## Material and Methods

Sixty patients with OA and concomitant Hp-associated GDP induced by NSAIDs were examined. Inclusion criteria were: the presence of clinical and radiological signs of OA, long-term use of NSAIDs, the presence of GDP caused by NSAIDs, the presence of Hp, and the informed consent of the patient to participate in the study. At the beginning and end of treatment, all patients were subjected to fibrogastroduodenoscopy with targeted biopsy according to the conventional method using an “Olympus” fibrogastroduodenoscope for the diagnosis of GDP. The presence of Hp was determined by invasive methods through a rapid urease test using samples from the biopsy obtained during endoscopy using the HELPIL®-test (“AMA”, St. Petersburg) diagnostic kit, morphological studies (staining with azure-II-eosin) and immunochromatographic test for the detection of Hp antigens in fecal samples (“Pharmasco” CerTest Biotec, SL, Spain). The content of epidermal growth factor (EGF) in blood plasma (set of reagents Human EGF (Hu EGF) ELISA Biosource, Belgium) and sAPO-1/Fas in serum (reagents Bender MedSystems, Austria) was determined by solid-phase enzyme-linked immunosorbent assay using the UNIPLAN analyzer, (AIFR-01) (PIKON, ZAO, Russia). TNF-α was studied in blood serum using a set of reagents (“ELISA-TNF-alpha”, Russia).

The basic therapy of OA included NSAIDs, non-narcotic analgesics, and chondroprotective drugs. Treatment of GDP induced by NSAIDs in patients with OA was performed according to the Maastricht Consensus, 2015. The presence of HP was determined 4 weeks after eradication therapy. Long-term effects were assessed at 6 months and 1 year after treatment.

Depending on the prescribed treatment, the examined patients were distributed as follows: Group I – 30 patients who received triple AHT together with primary OA therapy according to the Maastricht Consensus, 2015 (rabeprazole 20 mg twice per day for 28 days, clarithromycin 500 mg twice per day for 7 days, and amoxicillin 1000 mg twice per day for 7 days). In case of Hp resistance to standard triple therapy, second-line AHT was used (rabeprazole 20 mg twice a day for 28 days, bismuth tripotassium dicitrate 120 mg 4 times a day, metronidazole 500 mg 3 times a day, tetracycline 500 mg 4 times a day for 7 days). Group II consisted of 30 patients who took rebamipide (Mucogen, Macleods Pharmaceuticals Limited) along with AHT 1 tablet (100 mg) three times a day for 4 weeks.

## Results

Analysis of the obtained data showed that in patients with OA and concomitant Hp-associated GDP induced by NSAIDs, there is an imbalance between the physiological inhibitors and inducers of apoptosis (Table 1). Thus, in the examined patients before treatment, TNF-α increased by 4.71 (p<0.05) in group I and by 4.79 times (p<0.05) in group II compared with practically healthy persons (PHP). EGF also increased significantly – by 2.23 times (p<0.05) in group I and by 2.24 times (p<0.05) in group II compared with (PHP). Data on the reduction of sAPO-1/Fas evidenced an increase in the intensity of apoptosis processes in Hp-associated GDP caused by NSAIDs in patients with OA. Thus, in patients of groups I and II before treatment, the content of sAPO-1/Fas decreased by 3.89 (p<0.05) and 3.62 times (p<0.05), respectively, compared with PHP.

**Table 1.** The content of TNF-α, EGF and sAPO-1/Fas in Helicobacter pylori-positive gastroduodenopathies caused by NSAIDs in patients with osteoarthritis in the dynamics of treatment 1 month (M ± m).

The indicator studied	AHT (group I) n=30		AHT+ rebamipide group (group II) n=30		PHP n=20
	Before treatment	After 1-month treatment	Before treatment	After 1-month treatment	
**TNF-α, pc/ml**	34.73±3.78 p(H)<0.001	24.04±2.32 p(H)<0.001 p(BT)=0.019	35.33±3.94 p(H)<0.001	15.30±2.77 p(H)=0.027 p(BT)<0.001 p(R)=0.019	7.37±0.91
**sAPO-1/Fas, pc/ml**	62.03±8.40 p(H)<0.001	91.39±9.67 p(H)<0.001 p(BT)>0.05	66.68±9.29 p(H)<0.001	126.63±12.78 p(H) <0.001 p(BT)<0.05 p(R) <0.05	241.61±18.81
**EGF, pc/ml**	137.77±12.45 p(H)<0.001	164.82±10.38 p(H)<0.001 p(BT)>0.05	138.62±15.32 p(H)<0.001	196.29±11.96 p(H) <0.001 p(BT)=0.004 p(R) =0.052	61.79±9.04

n – the absolute number of patients; p(H) – the probability of disagreement with a group of virtually healthy individuals; p(BT) – the probability of disagreement with the group before treatment; p(R) – the probability of disagreement with the group where rebamipide is added to the AHT in the treatment regimen; AHT – antihelicobacter therapy; EGF – epidermal growth factor; PHP – practically healthy persons; TNF-α – tumor necrosis factor alpha.

After 1 month of treatment (Table 1), there was a decrease in apoptosis induction markers and an increase in protection, which indicates a positive effect of the prescribed treatment. Thus, the content of TNF-α in patients of group I decreased by 30.8% (p<0.05); in patients of group II, it decreased significantly by 56.7% (p<0.05) after additional inclusion in the treatment of rebamipide. In group II, the level of this cytokine was significantly reduced by 36.4% after treatment compared with this indicator in group I. The soluble form of APO-1/Fas increased in all groups after treatment. In group II, the content of sAPO-1/Fas significantly increased by 1.89 times after treatment. In all groups, there was an increasing trend of EGF, which can be explained by the ability of proton pump inhibitors (PPIs) to stimulate proliferation. However, these figures were not reliable in group I. There was a significant increase in EGF - by 1.42 times (p<0.05) in patients receiving rebamipide, which was significantly higher compared with group I. The long-term effects were assessed at 6 months and 1 year after treatment (Table 2). TNF-α tended to decrease during this period.

**Table 2.** The content of TNF-α, EGF and sAPO-1/Fas in Helicobacter pylori-positive gastroduodenopathies caused by NSAIDs in patients with osteoarthritis in the dynamics of treatment 6 and 12 months (M ± m)

The indicator studied	AGT (group I) n=30		AGT+ rebamipide group (group II) n=30		PHP n=20
	After 6-month treatment	After 1-year treatment	After 6-month treatment	After 1-year treatment	
**TNF-α, pc/ml**	20.95±2.25 p(H)<0.001	19.91±2.42 p(H)<0.05	11.09±2.09 p(R)=0.002	10.45±2.44 p(R)=0.008	7.37±0.91
**sAPO-1/Fas, pc/ml**	128.76±14.18 p(H)<0.05	166.85±12.19 p(H)<0.05	174.94±12.41 p(H)<0.05 p(R)=0.017	215.74±14.92 p(H)<0.05 p(R)=0.014	241.61±18.81
**EGF, pc/ml**	143.46±13.70 p(H)<0.05	124.35±11.51 p(H)<0.05	123.23±11.15 p(H)<0.05 p(R)>0.05	98.33±8.59 p(H)>0.05 p(R)>0.05	61.79±9.04

n – the absolute number of patients; p(H) – the probability of disagreement with a group of virtually healthy individuals; p(R) – the probability of disagreement with the group where rebamipide is added to the AHT in the treatment regimen; AHT – antihelicobacter therapy; EGF – epidermal growth factor; PHP – practically healthy persons; TNF-α – tumor necrosis factor alpha.

In particular, after 1 year, there was a decrease in the levels of cytokine by 17.2% (p>0.05) compared with this indicator after 1 month of treatment in group I. In patients receiving additional rebamipide, TNF-α was significantly reduced by 31.7% compared with data after 1 month of treatment and was significantly lower compared to group I. The soluble form of APO-1/Fas increased in all groups, but a more significant increase in this indicator was found in group II. EGF levels tended to decrease in all groups in the long-term treatment, but the data were not reliable. It is worth noting a significant decrease in EGF levels in the group where patients received additional rebamipide. Thus, EGF levels decreased by 1.99 times 1 year after treatment compared to the results obtained after only a month of treatment in patients of group II.

## Discussion

Under physiological conditions, a complex balance is maintained in the gastric mucosa between proliferation processes and programmed cell death. Hp is able to powerfully stimulate the proliferation of epithelial cells of the gastric mucosa. Simultaneously with the proliferation of Hp, it induces apoptosis in these cells by activation of the FasL/Fas signaling pathway [8], increasing cell death in the ulcer, which slows healing. By activating the production of cytokines, in particular TNF-α, Hp stimulates Fas-mediated apoptosis of epithelial cells. This cytokine is a potent inhibitor of gastric acid production. As a result, Hp colonization increases, and the inflammatory process progresses [9–12]. TNF-α is a key inflammatory cytokine that regulates proliferation, differentiation and initiates apoptosis. The biological effects of TNF-α depend on its concentration.

Increased levels of TNF-α in the examined patients indicate damage and inflammation. This proinflammatory cytokine is also secreted in early OA and triggers inflammation in chondrocytes and synovitis [13–15]. The bacterial peptidoglycan delivered from Hp stimulates the recognition of the cytoplasmic pathogen receptor in epithelial cells and induces inflammatory cytokines, including IL-1, IL-6, IL-8, TNF-α, and interferon-γ [16, 17].

NSAIDs also slow reparative regeneration, delay mucosal regeneration, and slow angiogenesis in granulation tissue. Neutrophilic infiltration of the regenerating epithelium, prostaglandin deficiency, and impaired angiogenesis interfere with healing. Inhibition of NSAID prostaglandin synthesis leads to stimulation of lipoxygenase pathway activation and increased leukotriene synthesis.

Leukotrienes cause inflammation and tissue ischemia, which leads to damage to the gastric mucosa. In addition, there is an increased production of proinflammatory mediators, such as tumor necrosis factors, which leads to a decrease in gastric blood flow and the release of free radicals. Oxygen-free radicals react with polyunsaturated fatty acids of the mucosa, which leads to lipid peroxidation and tissue damage. NSAIDs inhibit proliferation and dramatically increase the apoptotic activity of the epithelium [9, 18]. NSAIDs are also able to induce not only apoptosis but also necrosis in the gastric mucosa.

After 1 month of treatment, EGF tended to increase in all groups, which probably indicates towards compensatory protection. EGF accelerates tissue proliferation and epithelialization, binds to receptors on the surface of cell membranes, stimulates fibroblast taxis and epithelial cell differentiation, promoting wound healing [19, 20]. Contrary, when evaluating the long-term treatment, EGF decreased significantly.

The literature suggests that PPIs inhibit NSAID-induced Fas-mediated mucosal cell death by regulating decreased Fas or FasL expression and inhibiting caspase-8 activation, also contributing to the renewal of mucosal cells while stimulating the growth of epidermal growth factor and the main growth factor of fibroblasts and activates angiogenesis to restore the gastric mucosa [21]. Rebamipide (Mucogen) is a gastroprotective agent that stimulates the synthesis of prostaglandins in the gastric mucosa, increases the content of mucus glycoproteins, improves blood circulation to the mucous membrane of the digestive tract, and increases the expression of EGF and its receptors [22].

According to the presented data, the content of apoptosis induction markers decreases after treatment, and the indicators of protection increase, which indicates a positive effect of the prescribed treatment. In patients receiving AHT, these changes were minimal. The combination of standard treatment with rebamipide contributed to a significant improvement.

## Conclusions

In Helicobacter pylori-associated gastroduodenopathies caused by nonsteroidal anti-inflammatory drugs in patients with osteoarthritis, there is an increase in apoptosis intensity (increase in the serum levels of tumor necrosis factor-α with a simultaneous decrease in the content of sAPO-1/Fas) and an increase in the content of epidermal growth factor.

Inclusion of rebamipide in the complex 1-month treatment of Helicobacter pylori-associated gastroduodenopathies caused by nonsteroidal anti-inflammatory drugs in patients with osteoarthritis showed a significant decrease in tumor necrosis factor-α and compensatory growth of epidermal growth factor reparative processes in the mucous membrane of the stomach and duodenum.

In the long-term follow-up, after 6 months and 1 year of treatment of Helicobacter pylori-associated gastroduodenopathies caused by nonsteroidal anti-inflammatory drugs in patients with osteoarthritis, a significant decrease in tumor necrosis factor-α and epidermal growth factor on the background of sAPO-1/Fas growth with additional inclusion in the treatment of rebamipide in comparison with anti-Helicobacter therapy was seen, which helped to reduce the recurrence of gastroduodenopathies. We suggest using rebamipide to prevent gastroduodenopathies caused by nonsteroidal anti-inflammatory drugs in patients with osteoarthritis.

## Acknowledgments

### Ethical approval

The approval for this study was obtained from the Ethics Committee of the Bukovynian State Medical University (approval ID: 11-09-2016-318).

### Consent to participate

Written informed consent was obtained from the participants.

### Conflict of interest

The authors declare that there is no conflict of interest.
